# “It’s Only Brain Surgery”: Using 3D Printing and Simulation to Prepare Rural Physicians for the Management of Acute Epidural Hematoma

**DOI:** 10.7759/cureus.11236

**Published:** 2020-10-29

**Authors:** Dakotah Janes, Darrell Boone, Adam Dubrowski

**Affiliations:** 1 Medicine, Faculty of Medicine, Memorial University of Newfoundland, St. John's, CAN; 2 General Surgery, Memorial University of Newfoundland, St. John’s, CAN; 3 Health Sciences, Ontario Tech University, Oshawa, CAN

**Keywords:** burr hole, epidural hematoma, increased intracranial pressure, simulation

## Abstract

Patients presenting to rural emergency departments with increased intracranial pressure (ICP) can be challenging to diagnose, manage, and treat and although the presentation is rare, it is associated with high morbidity and mortality. In areas such as Newfoundland and Labrador, Canada, where the majority of the province is located far from tertiary care, this problem can be compounded by adverse weather impeding transport, necessitating that the problem is handled by rural physicians instead of neurosurgical care. However, many rural medical personnel do not receive any formal training in treating increased ICP. In this technical report, we use a low-tech, low-cost, high fidelity 3D printed skull to outline a simulation of increased ICP to better prepare rural physicians and emergency department teams who may encounter such a scenario in their practice in a rural area.

## Introduction

Head injuries and consequent traumatic brain injuries (TBIs) are among the most common types of trauma encountered in the emergency department [1]. The most important complication of traumatic brain injury is an intracranial hematoma, complicating 25% to 45% of severe TBI cases. The most dangerous but treatable complication is an acute epidural hematoma (also known as epidural bleeding), which is a potentially fatal collection of blood between the covering of the brain (the “dura”) and the skull. The condition can be fatal, as the buildup of blood increases intracranial pressure, compresses the brain tissue, and then causes “brain shift.” As many as 60% of cases are fatal [1].

Effective and immediate surgical management for acute epidural hematoma can transform a potentially rapid-death situation or potential permanent vegetative condition to a more benign clinical course with the expectation of recovery [2]. Rapid surgical management - the evacuation of the collection of blood by surgical decompression can be life-saving but is time-limited: evidence suggests that patients treated surgically within two hours of the loss of consciousness exhibited a mortality rate of 17% and good recovery in 67% as compared with a mortality rate of 56% and good results in 13% of patients operated on later [3]. However, the majority of the population (60%) of Newfoundland and Labrador, for example, are located farther than one hour away from a trauma centre (the highest proportion in Canada) and, moreover, 42.3% of the population of Newfoundland and Labrador live more than four hours away from neurosurgical care [4]. Accordingly, in rural areas, life-saving measures to relieve intracranial pressure may have to be carried out by non-specialists who are not residency-trained to “competency.”

Simulation-based education that includes deliberate practice has been shown to be superior to traditional medical education in achieving specific clinical acquisition goals [5]. Because there is a lack of exposure to acute epidural hematoma during residency, simulation-based training is a good alternative that could provide basic skills and knowledge so that this presentation can be treated in a more timely and proficient manner. This can be carried out on a low-tech, low-cost, high-fidelity 3D-printed skull simulator where a neurosurgeon or more experienced general surgeon can explain how to relieve the effects of an acute epidural hematoma. This could be in the form of burr hole placement or with a more complex procedure, such as decompressive craniotomy, depending on the situation and targeting group of learners. This technical report was created to provide rural physicians with a clinical scenario and a 3D-printed skull simulator to simulate a more realistic clinical encounter where they are required to manage an acute epidural hematoma to better prepare them should they ever be faced with this daunting task - actual brain surgery - in clinical practice. This simulation training can be, therefore, implemented into trauma training or it could be used in a live telesimulation for physicians located in rural areas. Hitherto, rural physicians train for burr hole evacuation of acute epidural hematomas by using coconuts or even cadavers and thus might stand to benefit from a higher fidelity model combined with a simulation. MacLellan, in a 1998 publication in the Canadian Journal of Rural Medicine, cited a story of a Society of Rural Physicians of Canada member in rural Alberta who evacuated an intracranial hematoma from a young child via faxed instructions from a neurosurgeon [6]. This anecdote led him to think about how fortunate this physician was to be able to contact a neurosurgeon and receive instruction. He then began to think about what he would do if he found himself in a similar situation with no guidance so he researched and published the required equipment list and instructions to get someone out of a bind should they be faced with the daunting task of surgical decompression of an intracranial hematoma in the periphery [6]. This technical report can be seen as an extension to MacLellan’s paper from nearly two decades ago. It provides us with a model to work on that is high fidelity but low cost as well as a simulation scenario to better prepare rural physicians should this situation arise. Surely, it is beneficial to not only have read the instructions but to have put them into action on a model, managed the simulation and received valuable feedback on your performance. Going through this simulation may subsequently improve outcomes for patients with an acute epidural hematoma in rural areas. Other medical professionals could participate in the simulation for their own training, team building and exposure to a rarely encountered medical emergency.

The emerging technology of 3D-printed models may offer significant advantages for training to meet this challenging condition. The limited literature in the field of simulation of surgical decompression of intracranial hematomas supports this approach as a potentially effective method of skills teaching [7]. However, there are very few models on the market and those that exist are extremely costly. For example, OpenSurgSim (OSS), a multi-platform open-source toolkit for the rapid development of real-time surgical training simulators is being used as a burr hole trainer, which is a much more advanced tool with didactics review and scenario selection, surgical simulation and metrics, but its development cost approximately eight million dollars [8]. Some models for treatment of aneurysms have been developed for about $600 but they are not developed for burr hole placements or craniotomies in particular [9]. A US-based company, Operative Experience, is the only commercial company selling 3D-printed models for personal use in the endeavour of medical training in hematoma decompression and costs approximately USD 3,000.

In this article, we present a detailed simulation scenario of a traumatic brain injury, using a low-cost 3D-printed skull simulator. In the simulation scenario, the patient arrives with increased intracranial pressure (ICP) following a motor vehicle accident under adverse conditions (heavy snowfall and raging winds), making an immediate transfer to neurosurgery impossible. The simulation can be given as “face-to-face” or via telemedical technologies (i.e. tele-simulation). Learning objectives are provided in the next section. The simulation fulfills the CanMEDS roles of Medical Expert, Scholar, Communicator and Collaborator, and after completion of this simulation, learners should have improved skills and confidence to manage increased ICP and increased competence to communicate and collaborate effectively in a team setting.

## Technical report

Table 1 lists the learning objectives of this simulation. 

**Table 1:** Learning objectives ABC: airway, breathing and circulation; ICP: intracranial pressure

Learning Objectives
1) Recognition of increased intracranial pressure and hypovolemia (included for the assessment/management of ABCs).
2) Medical management of increased ICP and performance of surgical evacuation/decompression, sterilizing and preparing the patient, selecting the correct instruments and performing burr hole placement (or any other form of decompression/surgical evacuation depending on the setting).
3) Communicating and collaborating effectively with a team in an emergency situation (assessed informally based on the evaluator’s observations and the feedback of team members in the debrief).

All the steps listed in this simulation-based scenario - the educational context, inputs, processes, and expected products related to the development and implementation of this simulation - are arranged according to a modified Context, Input, Process, and Product (CIPP) program development and evaluation model [10]. The management described in this technical report is based on one described by Gomersall et al. For details of the procedure and equipment, readers are encouraged to access the original source [11].

Context

The simulation described is meant for a team-based manikin scenario for the acquisition of technical skills on task trainers. The possible contexts for this simulation are in an emergency department, trauma bay, medical school, hospital-based simulation centre or even a rural hospital with access to tele-simulation equipment or a combination of these contexts. The main learner would be a rural physician or resident with nurses, medical students and other physicians to assist. The scenario described below can be tweaked to accommodate the available space, intended learners and the use of confederates and even the procedure - i.e. burr hole evacuation vs decompressive craniotomy (for rural general surgeons or residents, for example).

Inputs/equipment

**Figure 1:**
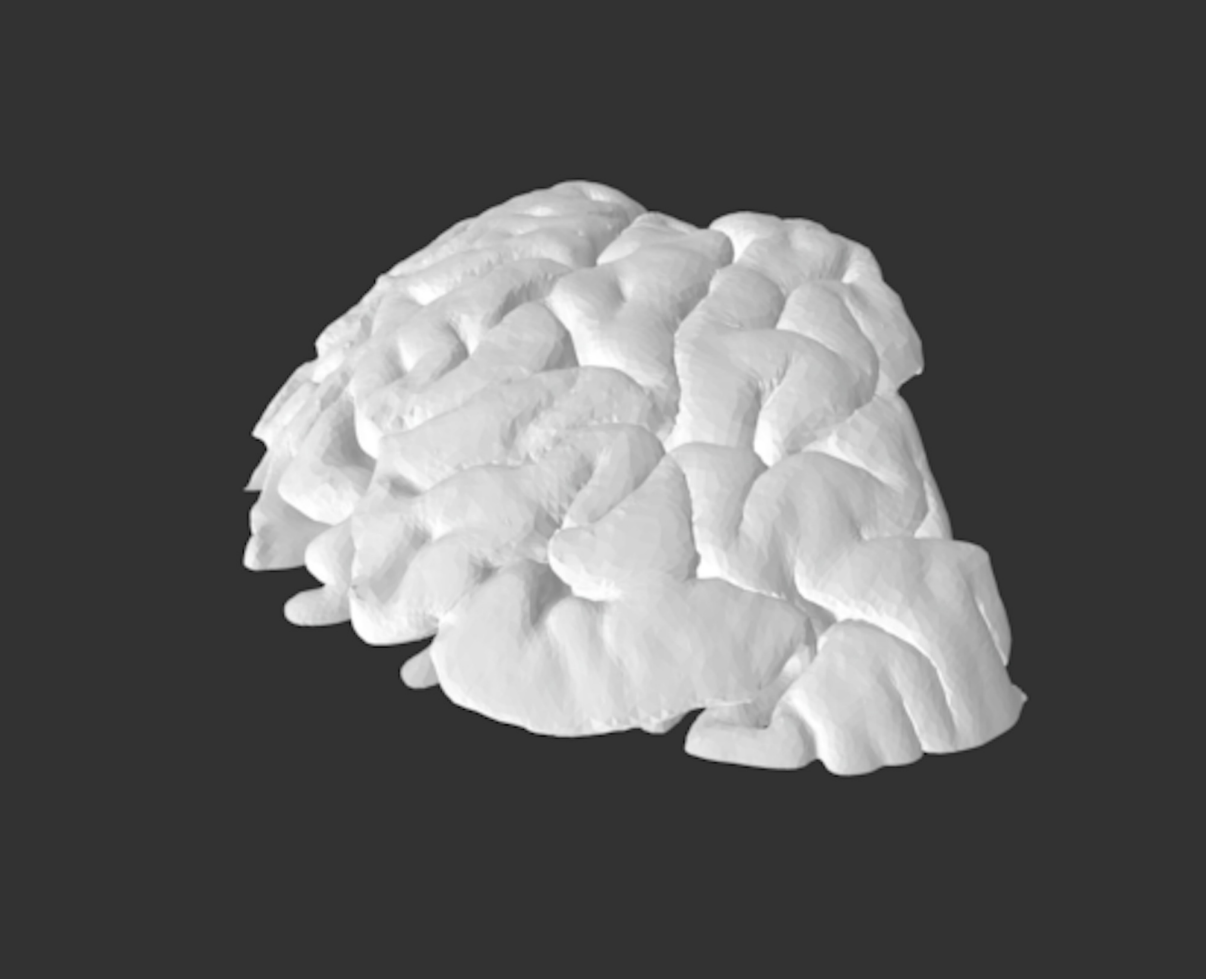
JPEG version of STI file used for 3D printing STI files available upon request

**Figure 2:**
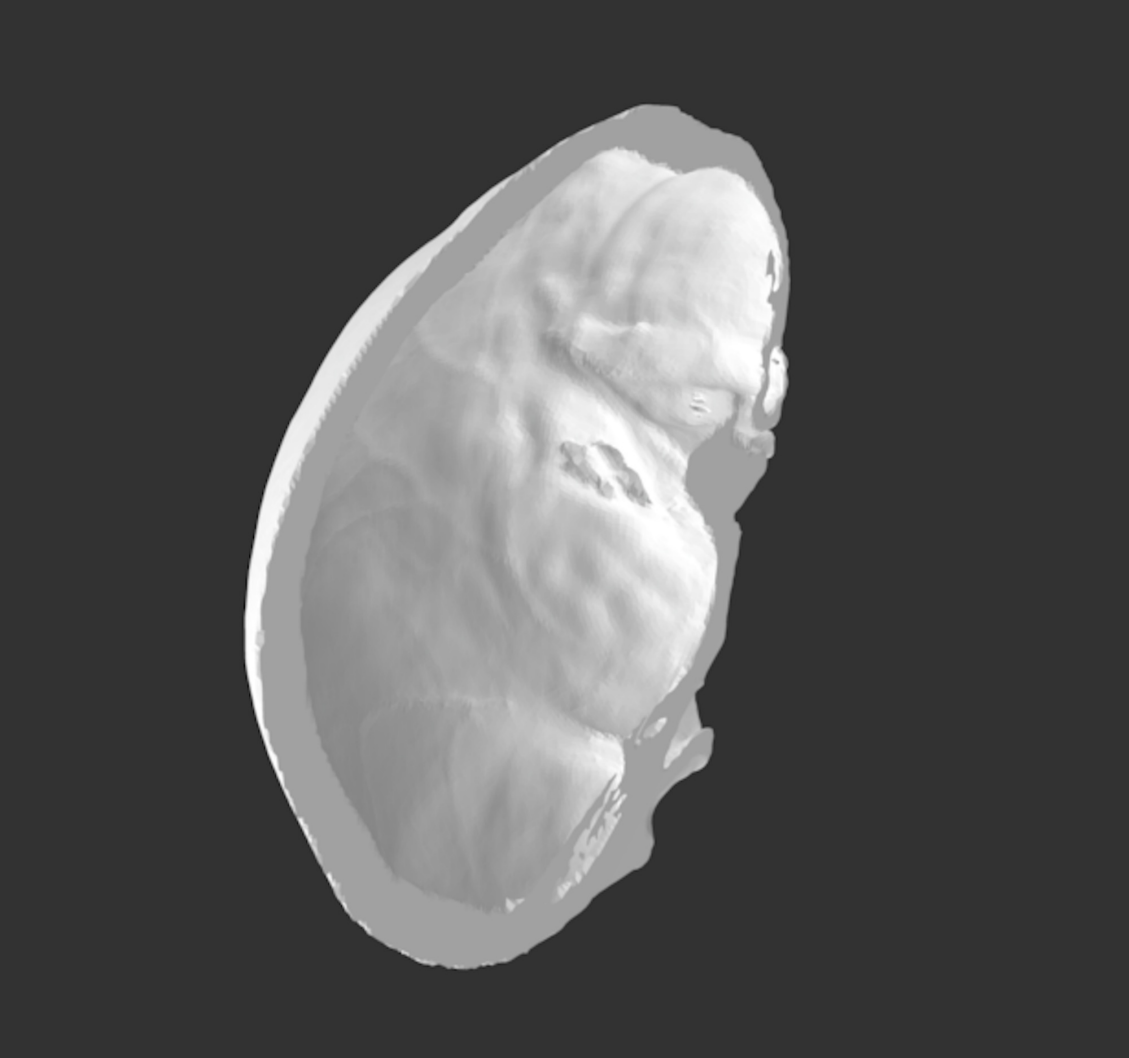
JPEG version of STI file used for 3D printing

**Figure 3:**
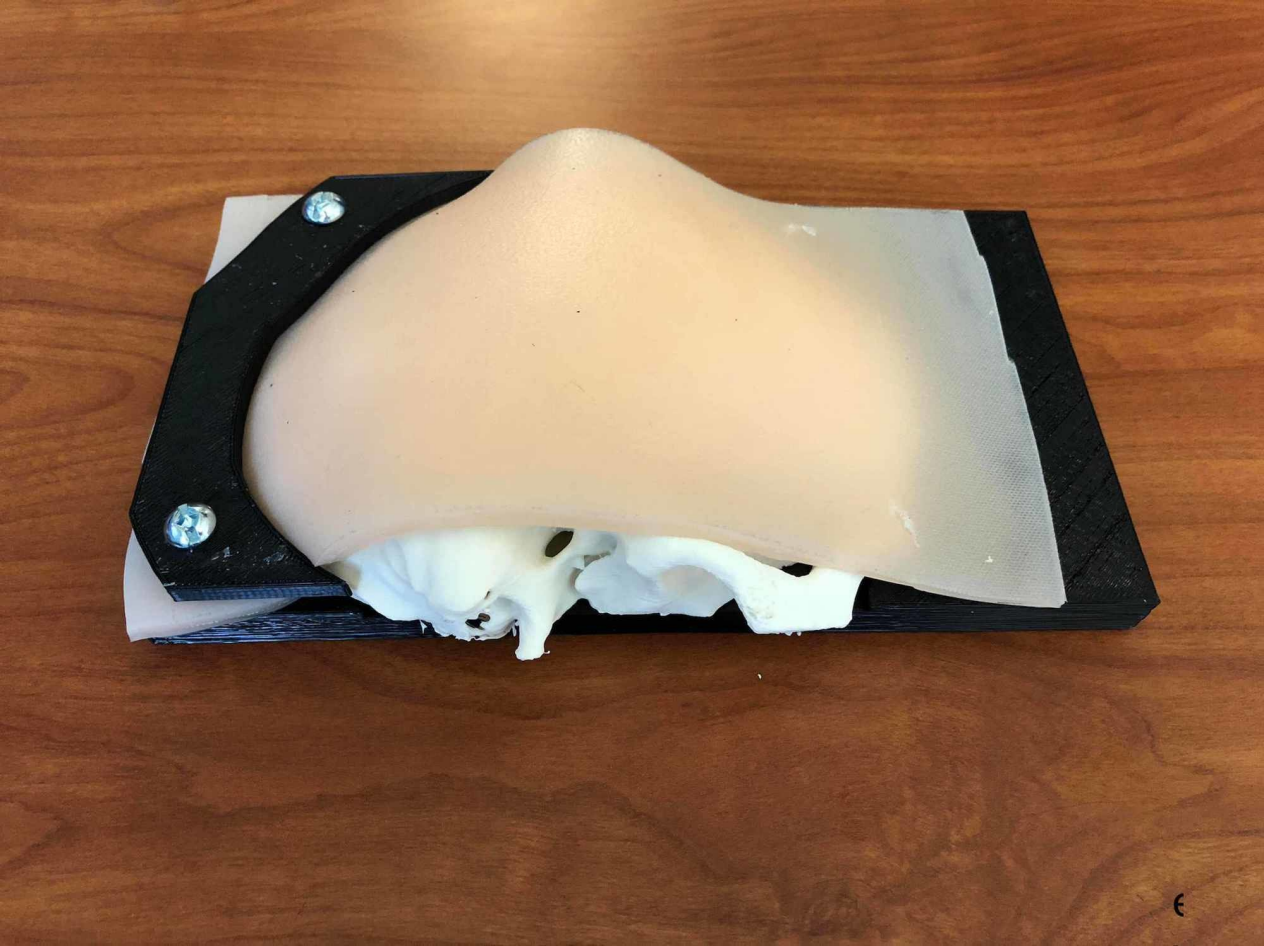
Intact craniotomy simulator

**Figure 4:**
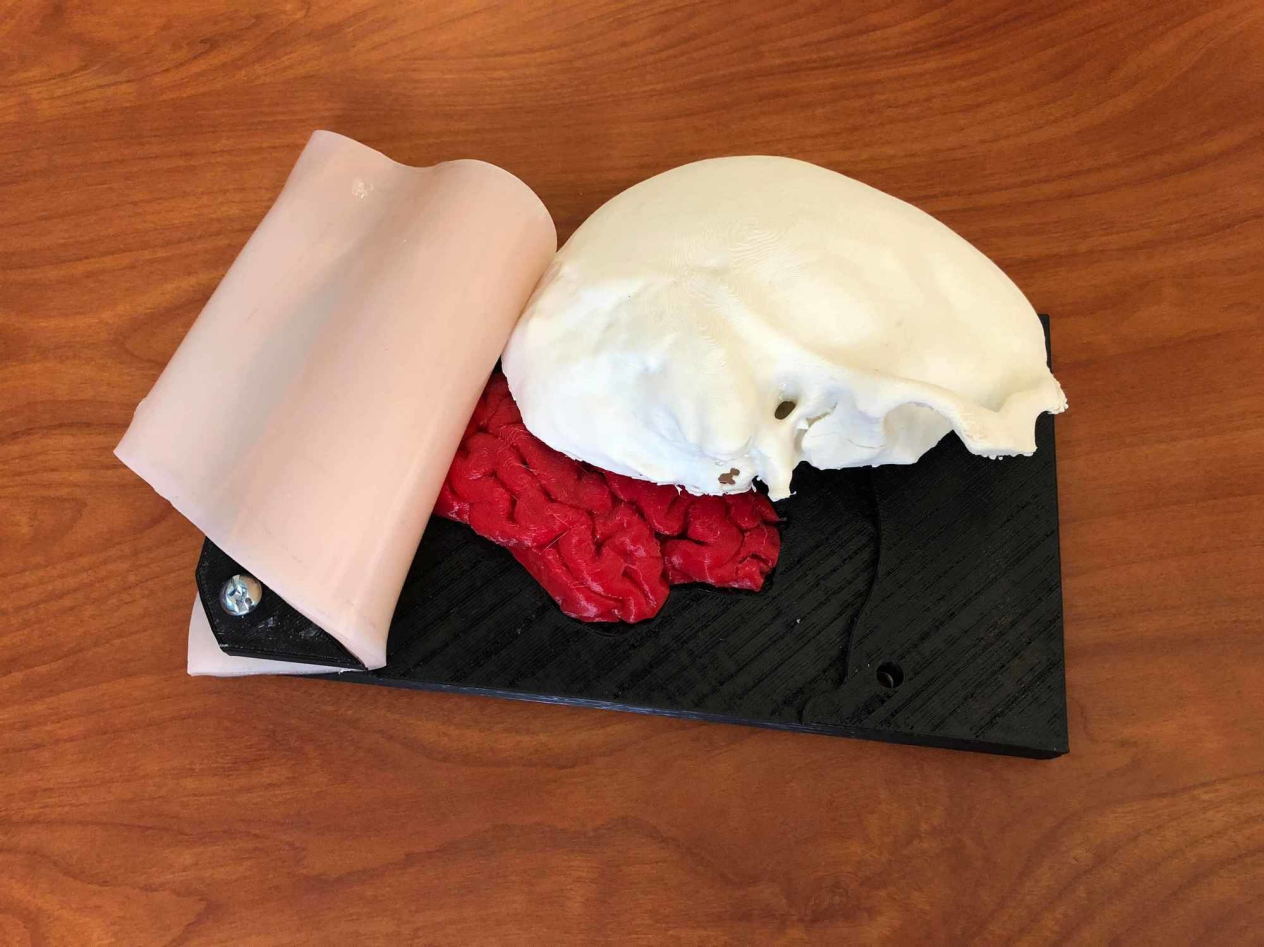
The craniotomy simulator with “skin” removed to show all three layers.

**Figure 5:**
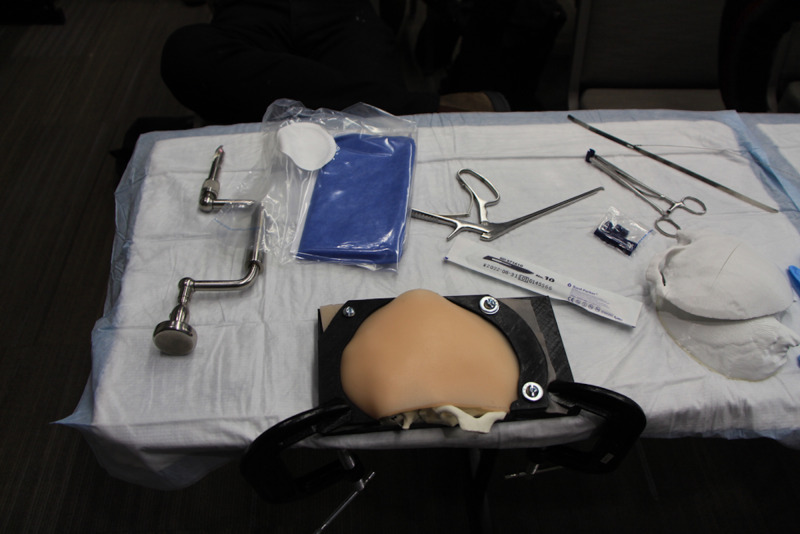
The craniotomy simulator and sample instruments for evacuation

Table 2 lists the equipment needed to run the simulation. Figures 1-5 show different aspects of the simulation.

**Table 2:** Inputs/equipment

Inputs/Equipment	
1) Skull simulator	A cost-efficient, low-tech, high-fidelity 3D-printed skull. The model consists of a skin and soft tissue layer of the skull, which is composed of 3 layers: silicone poured onto powder-mesh, a layer of gel to represent subcutaneous fat and a layer of silicone representing muscle. An anonymized head CT scan was converted to a 3D-printable file using Meshmixer and Osirix Lite (Figures 1-2). Low-density acrylonitrile-butadiene-styrene (ABS), an oil-based plastic, was used to approximate cranial bone density. The dura is composed of a thin layer of Ninjaflex material (a TPU-thermoplastic polyurethane-based material) and Ninjaflex material derived from an open-source file was used for the brain. The continuation of this model sees changes such as a larger surface area of the dura, so that it may be held in place by the skull, the skull is printed linearly rather than concentrically to prevent fracture during the simulation and the brain is derived from the same anonymized head CT to improve fit. This model costs $35.42 to print (Figures 3-4).
2) Instruments required to perform the evacuation of the epidural hematoma	This may include antiseptic skin preparation, scalpel, Raney clips and clip applicator, irrigation and syringe, Gigli Saw, artery clamp, forceps, Metzenbaum scissors, Hudson brace drill and bipolar cautery (Figure 5). Other equipment lists and step-by-step instructions are available depending on which method of surgical evacuation is chosen [6,12].

Process

The facilitator should have a printed copy of this report, the storyboard (Table 3) and the objectives to use as a guide for the simulation. There are also more detailed objective descriptions available to read in the report. The facilitator will designate the roles of the main learner and supporting team members prior to commencing the simulation. The scenario begins with the learner having the case read to them. When ready, the team will begin the simulation. As the simulation progresses, the facilitator should follow along on the storyboard and objective summary sheets. This simulation has two main objectives: recognition and medical management of increased intracranial pressure and evacuation of the hematoma. Thus, there are two storyboards and two debriefing sessions, as there are two separate checklists for learning objectives. Communication is a third minor objective that can be assessed informally, with no checklist based on the observations of the evaluator and feedback of team members. There is no further training required for the person carrying out the simulation other than an introduction to the 3D model.

**Table 3:** Simulation storyboard ER: emergency room; MVA: motor vehicle accident; CPR: cardiopulmonary resuscitation

Scenario Part One: You are a physician working in a rural ER. During a ferocious snowstorm, you receive a call about an MVA. The patient is a 56-year-old male who was ejected from the vehicle and has a known head injury. He is intubated en-route. Manage and treat this patient.		
Begin scenario – The learner is being called to assist with a trauma code in the ER. The patient arrives via an ambulance.		
Objective 1: Recognize a situation of increased intracranial pressure and the subsequent medical management of increased ICP.		
Vital signs: heart rate (HR): 90, blood pressure (BP): 90/50, decreasing to 65/30, respiratory rate (RR): controlled oxygen saturation (SaO2): 97%, transition time: 2 minutes	Expected Actions: 1. Initial resuscitation. Primary Advanced Trauma Life Support Survey. Circulation: Recognize the potential for cardiac arrest with mild tachycardia and attach to monitor/defibrillator. Recognize hypotension. Administer vasopressor such as norepinephrine intravenously (0.2 mcg/kg/min). Administer normal saline Intravenously (20cc/kg). Airway/breathing: controlled. Disability: since Glasgow Coma Score 8, check pupillary size and responsiveness.	Cues: Handover from nursing staff, summary of the case stated above along with vitals.
If completed, continue to Expected Actions 2; if only vasopressor administered, go to transient response to vasopressor. If no action is taken, go to No Action-PEA.		

Vital signs HR: 90 BP: 120/70, RR: Controlled, SaO2: 97%, Transition time: 30 seconds	Expected Actions 2: Initial improvement. Learners should continue with the Secondary Advanced Trauma Life Support Survey (systematic ‘top-to-toe’ examination).	Cues
Continue to Expected Actions 3		
Vital signs: HR: 40 BP: 230/150; SaO2: 97%; unilateral mydriasis; transition time: 3 minutes	Expected Actions 3 Coning: Learners should use mild controlled mechanical hyperventilation to try to decrease ICP by causing cerebral vasoconstriction (pCO_2_ 30 mmHg). Position the patient’s head at 15-30 degrees. Administer mannitol (1 g/kg rapidly by intravenous push 15-30 mins) and furosemide (0.5-1 mg/kg (or 40 mg) IV over 1-2 minutes; may be increased to 80 mg if there is no adequate response within 1 hour; not to exceed 160-200 mg/dose.	Cues: If the learner does not pick up on mydriasis in the secondary survey at the director’s cue confederate, will announce it after completing a survey.
If no action, continue to No Action - Death; if completed, go to resolution.		

Vitals: HR: 90, BP: 140/70, RR: controlled SaO2: 97%, transition time: 1 minute	Resolution: the patient is stabilized.	Cues: Once the leader has achieved the previous objectives, state the new set of vitals aloud. Subsequently, ask the leader about the future plan for the management of this patient. Once the plan has been verbalized, the facilitator may verbalize the conclusion of the simulation. Debrief of medical management of intracranial pressure.

Vitals: HR: 90, BP: 100/60, SAO2: 97%, transition time: BP increases over 30 seconds and goes back to 65/30 in 30 seconds (1 minute)	Transient response to vasopressor only	Cues: State change in vitals: HR dropping to 40 and BP increasing to 230/150
Go back to Expected Actions 3 after 30 seconds		

Vitals: HR: 90, BP: 40/35, RR/SaO2 - no trace, transition time: immediate	No action - Pulseless electrical activity: Learner must commence CPR and administer epinephrine intravenously (1 mg every 3-5 min). Administer normal saline intravenously (20 cc/kg).	Cues: If the learner is still missing hypovolemia, at the director’s cue, point out to the learner that the patient’s femur looks broken and that there must have been significant blood loss.
If completed, go to Expected Actions 2; if no action, continue to No Action - Death.		

Vitals: HR: 25, BP: 40/35, RR/SaO2: no trace, transition time: 1 minute	No action - Death. Learner announces the time of death.	Cues: Confederate verbalizes the conclusion of simulation and debriefs death.

Scenario Part Two: (only if medical management completed correctly): Patient from part one (56-year-old male involved in MVA with resulting increased ICP) has been in the ICU intubated and ventilated for 8 hours. He has been maxed out on medical management for high ICP and there is no improvement to vitals. The confederate (resident or another physician) contacts the neurosurgeon at the nearest tertiary care hospital who advises that the only treatment left for the patient is emergent surgical evacuation. Treat this patient.		
Begin Scenario: Learner arrives on the scene to task trainer prepped and draped. ER nurse on the scene to help.		
Objective 2: Complete surgical decompression/evacuation with the guidance of neurosurgeon via phone call or telesimulation.		
Vitals: HR: 55, BP: 170/90, RR Controlled SaO2: 97%, Transition time: 2 minutes	Expected Actions 1: The learner is guided through the surgical decompression on the task trainer by the neurosurgeon via tele simulation.	Cues: Confederate tells the learner that they will have to complete surgical decompression. They then ask if the resident knows how to perform this procedure. If yes, then continue with the procedure. If no, the neurosurgeon will walk them through it via telephone or tele-simulation.
Debrief: Surgical decompression successfully completed. Vitals: HR: 90, BP: 120/70, RR: Controlled, SaO2: 97%. Confederate goes through the checklist with the learner.		

Case

A 56-year-old male is brought in by emergency medical services (EMS) after being retrieved from the scene of a motor vehicle accident (MVA) at 3:00 pm. The province was experiencing a severe snowstorm, receiving 60 centimetres of snow over two days, combined with 80 km/hour winds. Reduced visibility and poor road conditions resulted in the accident. The patient was taken by EMS to the local emergency department. The emergency medical team and an ED nurse are immediately available with a lab and radiology technician available upon request.

The patient's wife provided a collateral history via telephone that revealed an allergy to penicillin, the only medication is omeprazole, and that he is a non-smoker with no pertinent past medical history. The patient was intubated en route. He has a known head injury.

The initial vitals were recorded as follows: heart rate: 100, blood pressure: 90/50 mmHg, oxygen saturation (SpO2): 97%, respiratory rate: controlled end-tidal carbon dioxide: 37.

Objective 1 (Simulation Part 1): Recognize a situation of increased intracranial pressure and the subsequent medical management of increased ICP.

With an initial assessment and complete set of vitals, the learner should go through the Primary Advanced Life Support Survey. They are then expected to develop an appropriate differential and treatment plan. A standard approach to primary assessment is expected, starting with airway, breathing and circulation (ABC). Abnormal vitals should be addressed at this stage. The ABCs are simplified in this simulation, as the patient was intubated en route. The main learning point here is to recognize tachycardia and the potential for cardiac arrest. They should also recognize hypotension and hypovolemia and, in turn, administer a vasopressor such as norepinephrine (IV 0.2 mcg/kg/min) and normal saline (IV 20 cc/kg). If this stage is completed effectively, the learner can continue to a period of initial improvement where they would continue with the Secondary Advanced Trauma Life Support Survey. If they administer only the vasopressor, they see transient improvement. If there is no action, the result is pulseless electrical activity (PEA). If PEA occurs, the learner will have to commence cardiopulmonary resuscitation (CPR) and administer epinephrine IV (1 mg every 3-5 minutes) along with IV normal saline (20 cc/kg).

The result of all actions is that the patient cones. The learner is expected to continue with medical management and use mild, controlled hyperventilation to try and decrease ICP by causing cerebral vasoconstriction. They should also try positioning the patient’s head at 15-30 degrees. The learner would then administer mannitol (1g/kg rapidly by IV push) and furosemide (0.5-1 mg/kg IV over 1-2 minutes). If the learner has not recognized the unilateral mydriasis by this point, the confederate will announce it at the director’s cue. If no action is taken at this point, the patient dies. However, if medical management is completed appropriately, the patient is stabilized and the conclusion of part one of the simulation is verbalized. There is a debrief and the facilitator goes through a checklist of the learner’s performance.

Objective 2 (Simulation Part 2): Evacuate the hematoma

In part two of the simulation, we imagine that it is eight hours later, and the patient has exhausted all options for the medical management of increased ICP and there is no improvement to vitals. A confederate such as a resident who is on service has contacted a neurosurgeon at the nearest tertiary care hospital who decides that because transport is not possible, surgical evacuation/decompression must be performed by the attending physician (learner). The learner arrives on the scene to the task trainer, prepped and draped, and with the guidance of the neurosurgeon via telesimulation and the help of the resident physician and nurse must complete surgical evacuation. Once the procedure is completed, there is a debrief and the facilitator goes through a checklist of the learner’s performance.

Products 

The expected products are organized according to the CanMEDS roles [13] and can be seen in Table 4:

**Table 4:** Products ICP: intracranial pressure

Products organized according to the CanMEDS roles
Medical Expert: The learner has to know how to recognize the presentation of increased intracranial pressure. The management of increased ICP involves clinical decision-making, interpreting vitals and then exhibiting procedural skill proficiency and learning novel skills in a stressful situation.
Scholar: The learner in this scenario is demonstrating a lifelong commitment to excellence in practice through continuous learning as is the neurosurgeon by teaching others along with the facilitators.
Communicator: The learner exhibits the communicator role through the appropriate use of closed-loop communication to ensuring effective team functioning.
Collaborator: The learner must work effectively with other health care professionals to provide safe, high-quality, patient-centred care in this simulation.

## Discussion

The aim of this simulation is to educate learners on how to approach and manage increased intracranial pressure in a rural hospital setting. Using a 3D-printed model is a cost-efficient way to provide hands-on training that could prove to be life-saving. The limited literature in this field addresses the fact that simulation-based learning for craniotomies is novel. Although this is a rather rare occurrence, it is so often fatal that it necessitates further training. Travel times paired with often harsh driving conditions and the time-sensitive nature of traumatic brain injuries make it obvious that at some time, someone will need this care and they will more than likely, need it performed where they are located, in a rural area. It is evident that for a surgical task, the most effective way to learn is through hands-on training. As this cannot be accomplished in real life, simulation is a great tool. The implications are improved surgical skills and improved quality of care for rural populations. As mentioned, the simulation is inexpensive and requires few resources and can be easily included in residency programs or at rural physician conferences.

Equipped with some hands-on experience in treating increased ICP rural physicians would be much more prepared to face one of the more challenging emergency situations where time is of the essence. This report provides a guideline for the management of increased ICP in settings where an experienced neurosurgeon is not available. Regardless of the learner’s level of training, after completing this scenario, the learner should have a better understanding of the approach to managing increased ICP as well as some complications that may arise. Moreover, the learner will receive feedback during a debriefing on their performance. This debriefing helps reinforce the correct technique, as well as identify areas in which they could improve.

The long-term goals of the simulation are for it to become part of residency training. The simulation could also be used as part of emergency medicine training, either for residents or practising physicians. As mentioned previously, cadavers and coconuts are currently being used for this purpose by rural physicians. Ideally, the training would be completed at least once but if deemed a suitable tool, it could become its own module that could be used alongside other certification training that is tested in four to five-year intervals such as Advanced Trauma Life Support (ATLS). This would ensure the retention of the acquired skills.

**Figure 6:**
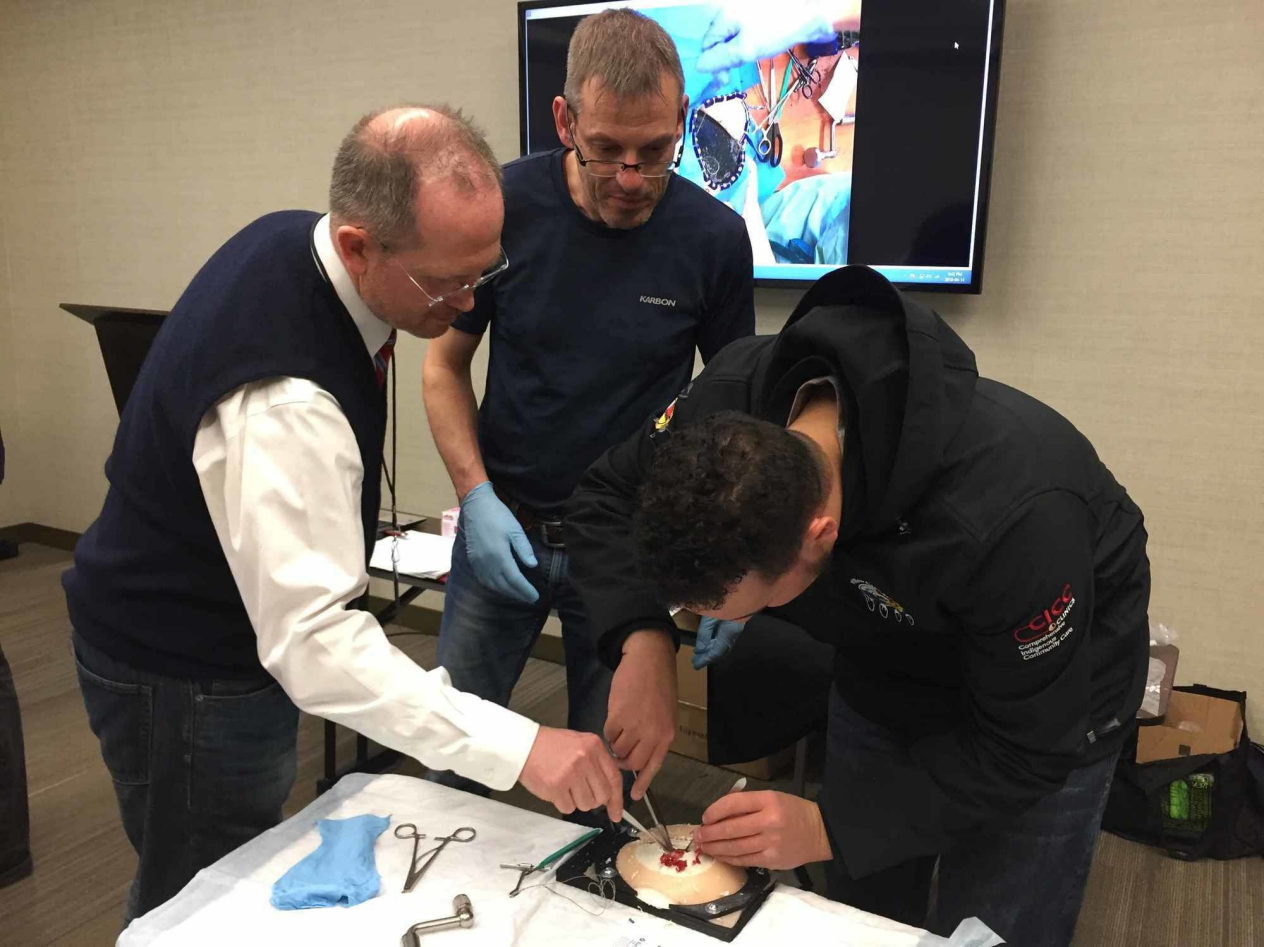
The 3D model being used for training purposes at the Society of Rural Physicians of Canada Conference in St. John’s, Newfoundland (April 2018)

As this is a novel simulation, there are no validated checklists. However, objective feedback systems, such as the Objective Structured Assessment of Technical Skills (OSATS) or a global rating scale, would be appropriate. The OSATS formally assesses discrete segments of surgical tasks using bench model simulations to assess technical skills [14]. The OSATS would be time-consuming and costly to implement, as there is no current OSATS tool for craniotomies but a Global Rating Scale (GRS) could be implemented. The GRS consists of seven dimensions, each related to some aspect of operative performance. Each dimension is graded on a five-point scale. Previous research has shown that this GRS is reliable and a valid assessment of technical skills both in the operating room and in the simulated operation [15]. If various levels of residents and experts try it and are graded using the GRS and the experts do very well and the residents not so well, it could have validity. The skull simulator was used at The 2018 Society of Rural Physicians of Canada Rural and Remote conference with great feedback (Figure 6). The main weakness of this simulation is that it is near impossible to test whether or not actually the simulation helps learners become more competent at the procedure as its occurrence is so rare, there is no way to quantify outcomes because of this training. However, if the simulation was tested by physicians with experience performing the procedure for realism (neurosurgeons or other), consistency, accuracy and reproducibility, that would be a good start as without the simulation, general surgeons will likely have no hands-on experience of the procedure before they may have to complete it in an emergency situation.

## Conclusions

Although rare, the complications of traumatic brain injuries, such as acute epidural hematoma, do occur. Although dangerous, it is treatable if effective and immediate surgical evacuation takes place. Patients living in rural areas are at increased risk of morbidity and mortality due to long travel times to neurosurgical care and travel is not infrequently exacerbated by inclement weather impeding transportation. Therefore, surgical evacuation may need to occur in the rural hospital where the patient presents. This may leave a rural physician facing a task that they have no exposure to and certainly have not been trained to competency in. This technical report provides learners with a simulation-based learning scenario, using a cost-efficient, high fidelity, 3D-printed skull model. It is a team-building simulation that can be tweaked to fit almost any low-resource situation to help train rural medical personnel for this potentially fatal condition. This simulation and 3D model could be used periodically for training purposes to ensure the retention of skills and help improve outcomes for patients.
